# Efficacy of metformin and electrical pulses in breast cancer MDA-MB-231 cells

**DOI:** 10.37349/etat.2024.00204

**Published:** 2024-02-19

**Authors:** Praveen Sahu, Ignacio G. Camarillo, Raji Sundararajan

**Affiliations:** Institute of Experimental Endocrinology and Oncology “G. Salvatore”-National Research Council (IEOS-CNR), Italy; ^1^School of Engineering Technology, Purdue University, West Lafayette, IN 47907, USA; ^2^Department of Biological Sciences, Purdue University, West Lafayette, IN 47907, USA; ^3^Purdue University Center for Cancer Research, Purdue University, West Lafayette, IN 47907, USA

**Keywords:** Metformin, triple-negative breast cancer, MDA-MB-231 cells, electroporation, glucose, reactive oxygen species, cell migration, wound healing assay

## Abstract

**Aim:**

Triple-negative breast cancer (TNBC) is a very aggressive subset of breast cancer, with limited treatment options, due to the lack of three commonly targeted receptors, which merits the need for novel treatments for TNBC. Towards this need, the use of metformin (Met), the most widely used type-2 diabetes drug worldwide, was explored as a repurposed anticancer agent. Cancer being a metabolic disease, the modulation of two crucial metabolites, glucose, and reactive oxygen species (ROS), is studied in MDA-MB-231 TNBC cells, using Met in the presence of electrical pulses (EP) to enhance the drug efficacy.

**Methods:**

MDA-MB-231, human TNBC cells were treated with Met in the presence of EP, with various concentrations Met of 1 mmol/L, 2.5 mmol/L, 5 mmol/L, and 10 mmol/L. EP of 500 V/cm, 800 V/cm, and 1,000 V/cm (with a pulse width of 100 µs at 1 s intervals) were applied to TNBC and the impact of these two treatments was studied. Various assays, including cell viability, microscopic inspection, glucose, ROS, and wound healing assay, were performed to characterize the response of the cells to the combination treatment.

**Results:**

Combining 1,000 V/cm with 5 mmol/L Met yielded cell viability as low as 42.6% at 24 h. The glucose level was reduced by 5.60-fold and the ROS levels were increased by 9.56-fold compared to the control, leading to apoptotic cell death.

**Conclusions:**

The results indicate the enhanced anticancer effect of Met in the presence of electric pulses. The cell growth is inhibited by suppressing glucose levels and elevated ROS. This shows a synergistic interplay between electroporation, Met, glucose, and ROS metabolic alterations. The results show promises for combinational therapy in TNBC patients.

## Introduction

Triple-negative breast cancer (TNBC) is a very aggressive subtype of breast cancer with limited treatment options [1–3]. The National Breast Cancer Coalition (NBCC) estimated more than 300,000 invasive breast cancer cases in women for 2023 [4]. Various advancements have been made in screening [5–7] and treatment methodologies, but cancer survival at the metastatic stage has not declined [8–12]. Around 15–20% of these breast cancer cases belong to the TNBC category [13]. Breast cancer gene 1 (*BRCA1*) and *BRCA2* are well-known mutations responsible for approximately half of the hereditary breast cancer and belong to the class of high-penetrance breast cancer susceptible genes [14–16]. TNBC is a *BRCA1*-related breast cancer phenotype [17]. TNBC are more aggressive phenotype with a high proliferation rate [18], and an overall 5-year relative survival rate of only 77%, while characterized by the lack of estrogen receptors (ERs), progesterone receptors (PRs), and human epidermal growth factor receptor 2 (HER2) [19, 20].

In TNBC, approximately 30 somatic mutation genes are significantly differentially expressed compared to non-TNBC [21]. These mutations are associated with tumor progression, stimulate cell proliferation, and promote cell survival via metabolite cell signaling pathways such as insulin-like growth factors (IGFs) signaling pathway [22], notch signaling [23], the nuclear factor-kappa B (NF-κB) signaling [24] and phosphoinositide 3 kinase (PI3K)/protein kinase B (AKT)/mammalian target of rapamycin (mTOR) signaling pathway [25], and mitogen-activated protein kinase (MAPK) signaling pathway [26, 27]. So, targeting the metabolic pathways involving metabolites presents a potential strategy for treating TNBC patients.

TNBC has a clinically high recurrence rate of 40%, issues like poor prognosis, and development of resistance to chemotherapy [28]. Due to the lack of canonical molecular targets in TNBC, treatment options are limited to conventional chemotherapy drugs [29, 30]. The lack of effective treatments motivated us to find an alternative combination of drug therapy. Towards this, the study explored the repurposing metformin (Met), the most administered type-2 diabetes drug, as an anticancer agent.

Met is a widely used antidiabetic drug [31] that is readily available worldwide and it is inexpensive [32]. It was first synthesized in the 1920s, and its potential antidiabetic properties were recognized in the 1950s [33]. It has also garnered attention for its possible benefits in cancer, polycystic ovary syndrome, and cardiovascular diseases [34, 35]. Its pharmacodynamic effects are through action on hepatic glucose production, peripheral glucose uptake, and insulin sensitivity [36]. It lowers the glucose released into the bloodstream by inhibiting gluconeogenesis [37, 38]. It promotes glucose transporter type 4 (GLUT4) translocation to the cell membrane, facilitating glucose uptake from the bloodstream into the cells [39, 40]. This action improves glucose utilization by peripheral tissues, lowering glucose levels. Met also has several other beneficial effects beyond its glucose-lowering actions [41]. It activates adenosine monophosphate (AMP)-activated protein kinase (AMPK), a cellular energy sensor that regulates various metabolic processes [42].

Met also exhibits antiproliferative and anti-inflammatory effects [43, 44], which may affect cancer prevention [45] and treatment [46, 47]. Prior studies show it can inhibit cell growth, induce cell cycle arrest, and promote apoptosis (programmed cell death) in cancer cells [48]. These effects are mediated through multiple pathways, including inhibition of mTOR signaling and modulation of AMPK activity [49, 50]. As seen earlier in TNBC, aberrant activation of mTOR signaling has been observed, leading to increased cell proliferation and survival. So, Met could target the mTOR pathway, and if the drug uptake is enhanced in the presence of electrical pulses (EP), it can lead to a more effective treatment for TNBC.

To enhance the intracellular Met concentrations, EP was utilized. This technique, known as electroporation, involves opening membrane pores that increase drug uptake when a combination of field intensity and sufficient pulse duration are applied [51–53]. The rate of pore density is given by Equation 1 [54], as shown below:

**Figure:**
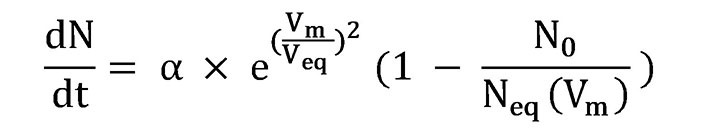


Here, α is a constant that depends on the properties of the cell membrane, V_eq_ is the voltage at which pores are created, V_m_ is the transmembrane potential, and N (t, θ) is pore density, θ is a tuning parameter that affects the relationship between N_eq_ and V_m_. N_eq_ is the equilibrium pore density for a given voltage V_m_, where N_0_ is the port density for V_m_ = 0 mV, and e is the base of the natural exponential (e = 2.71).

**Figure 1:**
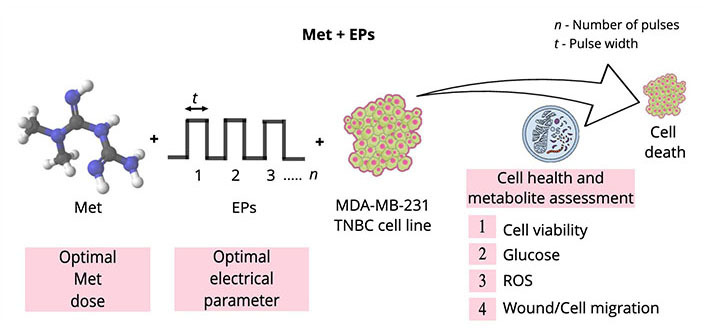
Illustration of the experimental design flow and the scope of the research work. Double-sided arrow: pulse width; ROS: reactive oxygen species

Electroporation-mediated chemotherapy, known as electrochemotherapy (ECT), has been extensively used in the European Union (EU) for treating cancer patients refractive to standard treatments. An extensive review by Joshi et al. [55] shows the successful implementation of ECT for various applications. Electroporation can minimize drug concentration and side effects on the surrounding tissues by improving drug efficacy [56]. Prior studies suggest that electroporation techniques can be effectively combined with various drugs to improve therapeutic efficacy [57–61]. This research examined the efficacy of a combination of Met with EP using various assays. The experimental design flow and the scope of the study are depicted in Figure 1.

## Materials and methods

### TNBC cell line

**Figure 2:**
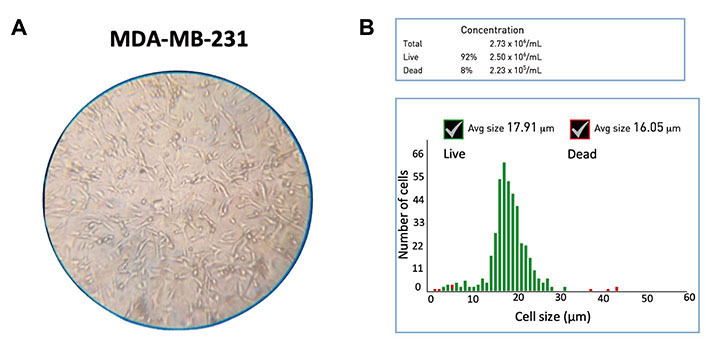
Characterization of MDA-MB-231 cell line. (A) Microscopic image (10×); (B) the Gaussian distribution for the cell diameter (mean: 18 µm) of the untreated MDA-MB-231 cells. Avg: average

MDA-MB-231 cell line (HTB-26^TM^, American Type Culture Collection^®^, USA) [62, 63] was used, which was originally derived from a 51-year-old white American woman with metastatic breast cancer [64]. MDA-MB-231 cells are spindle-like in shape with a mean diameter of 18–20 µm [65], as shown in Figure 2A and B. They were grown in the Dulbecco’s Modified Eagle Media (DMEM, 11965084, Thermo Fisher, USA) containing 10% fetal bovine serum (‎FBS, A5256801, Thermo Fisher, USA) and 1% penicillin-streptomycin (PS, 15070063, Thermo Fisher, USA) [66] to a final concentration of 10^6^ cells/mL.

### Met drug

**Figure 3:**
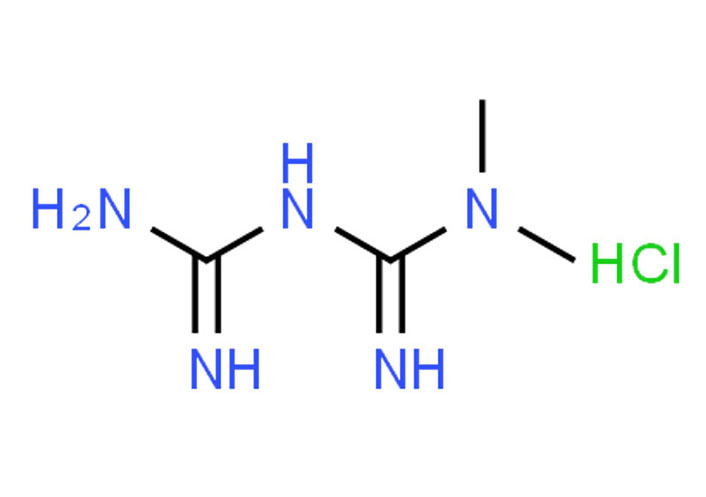
Chemical composition and structure of Met hydrochloride [76]

Met, approved by the Food and Drug Administration (FDA) [67], belongs to the biguanide class with two methyl functional groups (-C=NH-NH_2_) (Figure 3) [68–71]. It is an antidiabetic drug that shows antihyperglycemic activity (the ability of a substance to lower blood sugar levels) [72]. It is available as Met hydrochloride (1,1-dimethyl biguanide hydrochloride, C_4_H_12_ClN_5_) from Sigma Aldrich^®^ [73], a pharmaceutical reference standard. Its molecular weight is 165.62 g/moL, and its solubility is 200 mg/mL in water [74]. A long-term (10-year) study by the diabetes prevention program research group determines that Met is safe and well tolerated [75]. The concentrations for dose optimization and further studies on the MDA-MB-231 cell line used are 1 mmol/L, 2.5 mmol/L, 5 mmol/L, and 10 mmol/L.

### Electroporation

A BTX-ECM 830^®^ electroporator (Genetronics Inc. San Diego, USA) [77] was used to apply eight unipolar square pulses of 500 V/cm, 800 V/cm, and 1,000 V/cm, each with 100 µs pulse width for 1 s intervals. For electroporation, the cuvette having a 4 mm gap between the electrodes was filled with 600 µL of MDA-MB-231 cell suspension at 10^6^ cells/mL concentration adjusted to varying Met concentrations. The cuvette was inserted in the chamber, and the desired pulsing was applied. Following electroporation, cells were incubated in 96-well plates for various assays.

### Cell viability assay

Real-time-Glo™ MT cell viability assay (G9711, Promega, USA) was used, which provides viability without damaging the cells with a bio-luminescent signal output [78]. The treated samples were incubated in 96-well plates. Each well contains 20 µL (20,000 cells per well) of treated sample and 55 µL of cell media. MT cell viability substrate and nanoLuc enzyme were added in the ratio 1,000:4 and 25 µL was added to each well at t = 0 h. Here, the MT cell viability substrate reduces to form nanoLuc substrate after diffusing into cells. The nanoLuc substrate exiting the cell is rapidly used by nanoLuc luciferase in the media. The substrate is reduced only by metabolically active cells. Thus, the luminescence value indicates the number of live cells represented by Equation 2 (RLU means relative luminescence units), as shown below:

**Figure:**
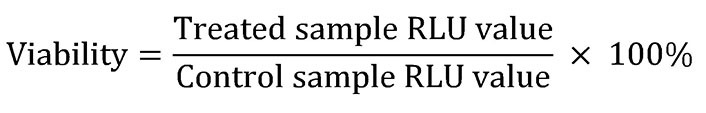


### Microscopic cell inspection

Microscopic images assisted in establishing a visual correlation of the chemical assay with the morphological change and effects of drugs on cancer cells [79]. The sample treatment includes control, 1,000 V/cm, 5 mmol/L Met, and 1,000 V/cm + 5 mmol/L Met. After treatments under these conditions, 100 µL (100,000 cells per well) were seeded in a 12-well plate. After 24 h of treatment, a microscope (M150^®^, AmScope, USA) was used to capture cell morphology transformations and mark any visible changes. The microscope was set to a 10× zoom to capture a more extensive field area. The acquired images were then analyzed, allowing to make inferences related to parameters such as cell morphology and co-localization.

### Glucose assay

Glucose Uptake-Glo™ assay (J1341, Promega, USA) is a homogeneous metabolite detection assay that quantifies the rate at which the glucose uptake transpires inside the cancer cell [80]. The sample treatment includes control, 1,000 V/cm, 5 mmol/L Met, and 1,000 V/cm + 5 mmol/L Met. After treatment, 20 µL (20,000 cells per well) was transferred to a 96-well plate, and 80 µL of cell culture media (DMEM) was added. Next, 50 µL of a prepared 5 mmol/L solution of 2-deoxy-*D*-glucose (2DG) was added to each well, followed by a gentle mix for 10 min before incubating the sample. After an incubation period of 23 h, the stop buffer (25 µL) was added to each well, followed by 25 µL of neutralization buffer, and gently mixed. Subsequently, 100 µL of 2-deoxyglucose-6-phosphate (2DG6P) detection reagent was added and briefly shaken. The 2DG6P detection reagent was prepared 1 h in advance to minimize the assay background. The 96-well plate was then incubated at room temperature for another 30 min. Finally, the RLU were recorded using Synergy HTX multi-mode microplate reader (SLXATS, BioTek Instruments, USA) at 24 h with a 0.5 s integration time. The luminescence values were normalized with respect to the control.

### ROS assay

ROS-Glo™ H_2_O_2_ assay (G882B, Promega, USA) was used to detect hydrogen peroxide (H_2_O_2_) in the sample directly, using a simple two-reagent protocol [81]. The sample treatment includes control, 1,000 V/cm, 5 mmol/L Met, and 1,000 V/cm + 5 mmol/L Met. After the treatment, cells were seeded in a 96-well plate at a desired density of 20,000 cells per well. An additional 60 µL of the medium was introduced to each well, making the final volume 80 µL. The cells were then placed in an incubator at 37°C CO_2_ incubator for 18 h. Next, the H_2_O_2_ substrate dilution buffer thawing was carried out gently and kept on ice. The H_2_O_2_ substrate solution was prepared by diluting the 10 mmol/L H_2_O_2_ substrate to 125 µmol/L in the H_2_O_2_ substrate dilution buffer, ensuring optimal mixing. Enough H_2_O_2_ substrate solution was prepared for all samples. At 18 h, 20 µL of the prepared H_2_O_2_ substrate solution was added to each well. This resulted in a final well volume of 100 µL and a final H_2_O_2_ substrate concentration of 25 µmol/L. The plate was incubated for the next 6 h. Following this, 100 µL of ROS-Glo™ detection solution was added per well. The plate was incubated at room temperature (22°–25°C) for 20 min, and the luminescence values were recorded using Synergy HTX multi-mode microplate reader (SLXATS, BioTek Instruments, USA) in RLU. The luminescence values were normalized with respect to the control for analysis.

### Wound healing assay

A wound healing assay was used to study the cell migration [82] of MDA-MB-231 cells under the influence of Met and electric pulse. The sample treatment includes control, 5 mmol/L Met, and 1,000 V/cm + 5 mmol/L Met. The culture-insert 2-well from Ibidi^®^ was used to create a defined cell-free gap of 500 µm in width [83]. The 10 mm culture dish was sterilized, and the culture-insert 2-well was inserted, creating two individual wells separated by a central divider. After treatment, 10 µL (10^6^ cells/mL) of cells were seeded to either side of the well. Then, the cells were allowed to grow for around 24 h inside the incubator (37°C, 5% CO_2_, and 85% relative humidity).

The culture insert was removed after 90–95% confluency, creating a highly reproducible cell-free gap or “wound” between 2 wells, as shown in step 2 (Gap-Creation). Ultimately, it was expected that the cells on the sides to close the gap by growing and migrating. EVOS-FLTM digital microscope (AMEFC4300, Invitrogen™, USA) was used to capture images and measure the initial gap (t = 0 h). Then, the cells were incubated, and after 24 h incubation, another image was taken to capture cell migration.

ImageJ (version 1.53i), an open-source image analysis software by the National Institutes of Health (NIH), was utilized to quantify the extent of wound closure by measuring the area of the cell-free gap [84] at t = 0 h and t = 24 h. This allowed for the quantitative measurement and assessment of the migratory capabilities of the MDA-MB-231 cells under the influence of Met and electric pulse.

### Statistical analysis

Repeated measure analysis of variance (ANOVA) was used to study the statistical significance of the assays, including cell viability, glucose, and ROS assay. Tukey’s test for multiple comparisons [85] was conducted after ANOVA. The significance level “*P*” was set to 0.05 to determine whether the difference between the means of any two-sample treatment is statistically significant. Also, after calculating the honestly significant difference (HSD) or the critical value (CV), alphabets (“A”, “B”, etc.) were assigned to all treatments. The Tukey’s HSD formula is given by Equation 3 [86], as shown below:

**Figure:**
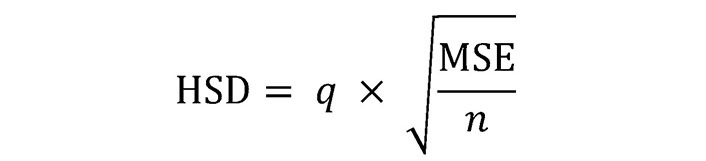


Here, *q* is the CV derived from the studentized range distribution, MSE is the residual mean square from ANOVA, and *n* is the number of observations in each group. The standard *q*-score Tukey’s table was used to obtain the *q*-value corresponding to the specific number of treatments (k), degree of freedom (Df) within treatments, and *n* = 3 (each of the sample treatments was performed in triplicate). Here, the same alphabets for the treatments indicate that they are not significantly (ns) different, i.e., the difference between treatments was less than that of HSD, whereas different alphabets (*P* < 0.05) indicate they differ significantly (s). The statistical analysis was done using R-studio. All the values are represented as mean ± standard error (*n* = 3).

## Results

### Dose curve

**Figure 4:**
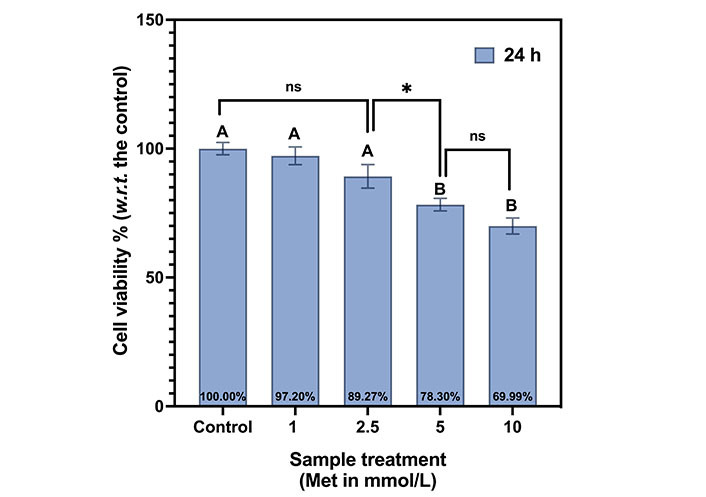
Met dosage curve for MDA-MB-231 cells at 24 h. The same letters indicate ns difference between the treatments, whereas different letters indicate a significant difference (^*^ *P* < 0.05). *w.r.t.*: with respect to

The dose curve at 24 h is shown in Figure 4. Here control is normalized to 100%. At 1 mmol/L, cell viability is 97.2% (slightly reduced compared to control), and it is 89.27% at 2.5 mmol/L. It declined to 78.3% at 5 mmol/L and 70% at 10 mmol/L. With the doubling of the drug dose from 5 mmol/L to 10 mmol/L, the reduction in viability is only 8.31%. This illustrates the saturation of the drug doses, and 5 mmol/L was chosen as the optimum dose for further experiments.

Tukey’s test indicates the same letter “A”, for control, 1 mmol/L, and 2.5 mmol/L, indicating that their viabilities are not statistically different (ns). The viabilities at both 5 mmol/L and 10 mmol/L are statistically different from the first three samples but not with each other. Thus, both are represented by the letter “B”. The summarizes Tukey’s test multiple comparisons between the samples in the Met dosage curve for MDA-MB-231 cells at 24 h is shown in Table 1.

**Table 1:** Summary of Tukey’s multiple comparisons for Met dose-curve in MDA-MB-231 cells at 24 h

**Tukey’s multiple comparisons**	**Mean difference**	**95% CI of difference**	**Above HSD**	**Summary**	**Adjusted *P* value**
Control *vs.* 1 mmol/L	2.8	–6.04 to 11.64	No	ns	0.8306
1 mmol/L *vs.* 2.5 mmol/L	7.93	–0.91 to 16.77	No	ns	0.0845
2.5 mmol/L *vs.* 5 mmol/L	10.97	2.12 to 19.81	Yes	*	0.0147
5 mmol/L *vs.* 10 mmol/L	8.31	–0.53 to 17.15	No	ns	0.068

^*^ *P* < 0.05. CI: confidence interval

### Electrical parameter optimization

**Figure 5:**
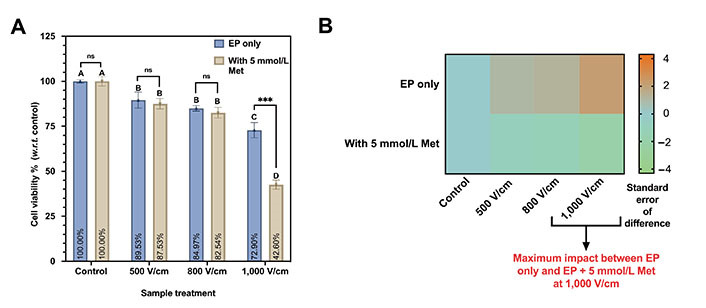
Cell viability assay on MDA-MB-231 at different EP conditions (500 V/cm, 800 V/cm, 1,000 V/cm, 100 µs, 1 s interval). (A) Viability percentage for EP only and EP + 5 mmol/L Met. The same letters indicate ns difference between the treatments, whereas different letters indicate a significant difference (^***^ *P* < 0.001); (B) the heatmap plot depicting the statistical difference in cell viability observed between EP only and EP + 5 mmol/L Met counterpart. *w.r.t.*: with respect to

The viabilities of the MDA-MB-231 cells with EP only and EP + 5 mmol/L conditions are shown in Figure 5A. The control is normalized to 100%. Under EP-only conditions, at 500 V/cm, the cell viability is 89.53% and 84.97% for 800 V/cm. The cell viability reduces as we increase the electric field. It is as low as 72.90% at 1,000 V/cm.

Further, Tukey’s test indicates ns difference was observed for 500 V/cm and 800 V/cm, and both were represented by the letter “B”. The viability at 1,000 V/cm (represented by the letter “C”) significantly differs from all other sample treatments.

Likewise, the cell viability for 500 V/cm and 800 V/cm with 5 mmol/L is 87.53% and 84.97%, respectively. It is a minor reduction of 2.56% in viability compared to a 60% increment in the electric field magnitude. However, 1,000 V/cm with 5 mmol/L Met shows a significant decrease in viability to 42.60%.

Based on Tukey’s test (Table 2), 500 V/cm and 800 V/cm with 5 mmol/L Met were not statistically significant, and both were represented by the same letter “B”. The difference in cell viability between 1,000 V/cm with 5 mmol/L is significantly different and represented by the same letter “D”.

**Table 2:** Summary of Tukey’s test for Met treatment with and without EP on MDA-MB-231

**Sample treatment**	**Above HSD**	**Summary**	***P* value**
500 V/cm *vs.* 500 V/cm + 5 mmol/L Met	No	ns	0.549472
800 V/cm *vs.* 800 V/cm + 5 mmol/L Met	No	ns	0.277932
1,000 V/cm *vs.* 1,000 V/cm + 5 mmol/L Met	Yes	***	0.000441

^***^ *P* < 0.001

The heatmap comparing the EP only and EP + 5 mmol/L Met groups with color code representing the standard error of the difference between both groups is shown in Figure 5B. A maximum was observed impact at 1,000 V/cm on viability with 5 mmol/L Met compared to the 1,000 V/cm counterpart.

### Microscopic cell inspection

**Figure 6:**
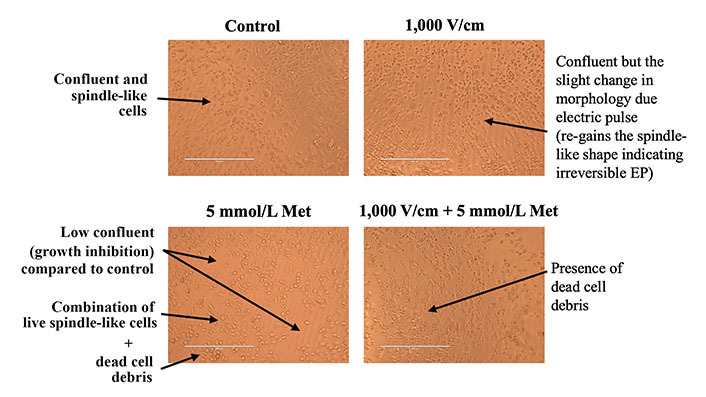
Microscopic image inspection of MDA-MB-231 cells at 24 h for control, 1,000 V/cm, 5 mmol/L Met and 1,000 V/cm + 5 mmol/L Met

The microscopic images for control, 1,000 V/cm, 5 mmol/L Met, and 1,000 V/cm + 5 mmol/L Met after 24 h of treatment are shown in Figure 6. In the case of control, cells appeared confluent and exhibited a spindle-like shape (characteristic of untreated MDA-MB-231 cells). At 1,000 V/cm, cell confluency was slightly lower compared to the control, with morphological changes induced by the electric pulses [87]. The 5 mmol/L Met sample exhibits a much lower cell confluence than the control, suggesting growth inhibition. It has a combination of live spindle-like cells and dead cell debris, indicating a mixed population of viable and non-viable cells.

Further, at 1,000 V/cm + 5 mmol/L Met, the presence of sizeable dead cell debris suggests an increased cytotoxic effect compared to the 5 mmol/L Met treatment counterpart. Thus, the combination of 1,000 V/cm with 5 mmol/L Met shows an enhanced cytotoxic effect.

### Glucose assay

**Figure 7:**
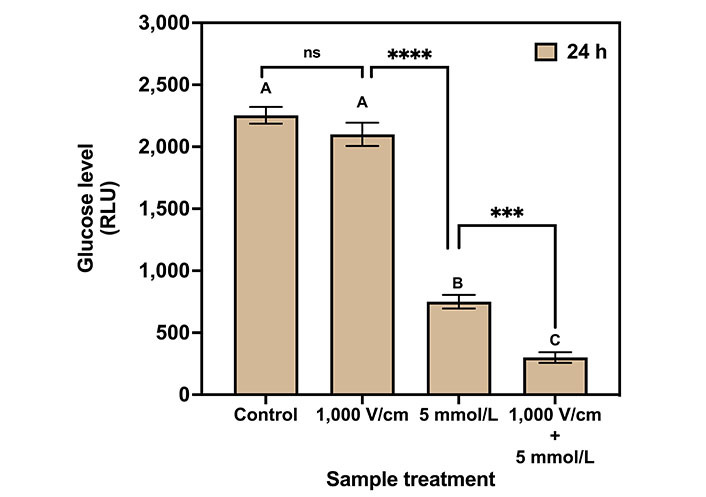
Glucose assay for various treatments on MDA-MB-231 cells after 24 h of treatment. The same letters indicate ns difference between the treatments, whereas different letters indicate a significant difference (^***^ *P* < 0.001; ^****^ *P* < 0.0001)

The results of the glucose assay for control, 1,000 V/cm, 5 mmol/L Met, and 1,000 V/cm + 5 mmol/L Met after 24 h of treatment are shown in Figure 7. The glucose levels are measured in RLU. The glucose level for the control sample was 2,253.78 RLU. At 1,000 V/cm, it was 1,919.75 RLU indicating a 14.82 % decrease in glucose level compared to control. For 5 mmol/L Met, the glucose levels were substantially reduced to 66.72% to 750 RLU compared to the control. Next, the combination of 1,000 V/cm with 5 mmol/L Met resulted in a glucose level of 400 RLU (5.6 times lower than the control), indicating a drastic reduction in glucose.

Tukey’s multiple comparisons test for glucose assay (Table 3) indicates no statistical significance between control and 1,000 V/cm hence represented by the same letter “A”. The 1,000 V/cm + 5 mmol/L Met is statistically different (1.9 times lower) compared to the 5 mmol/L Met counterpart and represented by the letters “C” and “B”, respectively.

**Table 3:** Summary of Tukey’s multiple comparisons test for glucose assay in MDA-MB-231 at 24 h

**Tukey’s multiple comparisons**	**Mean difference**	**95% CI of difference**	**Above HSD**	**Summary**	**Adjusted *P* value**
Control *vs.* 1,000 V/cm	154	–18.71 to 326.8	No	ns	0.0816
1,000 V/cm *vs.* 5 mmol/L Met	1,350	1,177 to 1,522	Yes	****	< 0.0001
5 mmol/L Met *vs.* 1,000 V/cm + 5 mmol/L	450	277.3 to 622.7	Yes	***	0.0001

^***^ *P* < 0.001; ^****^ *P* < 0.0001

### ROS assay

**Figure 8:**
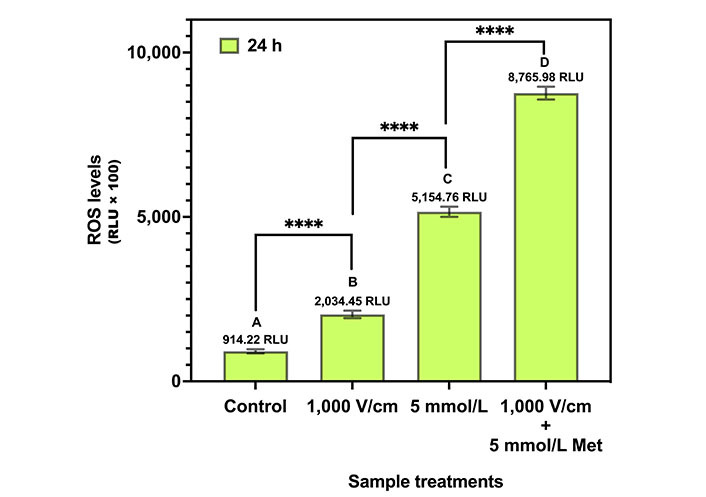
ROS assay for various treatments in MDA-MB-231 cells at 24 h. The same letters indicate ns difference between the treatments, whereas different letters indicate a significant difference (^****^ *P* < 0.0001)

The result of the ROS assay for control, 1,000 V/cm, 5 mmol/L Met, and 1,000 V/cm + 5 mmol/L Met after 24 h of treatment is shown in Figure 8. The H_2_O_2_ levels are represented as RLU × 100. The control exhibited a ROS level of 914.22 RLU. For 1,000 V/cm, a 2.2-fold increase in ROS production was observed compared to the control. In the case of 5 mmol/L Met, ROS levels increment by 5.6-fold compared to the control. Further, for 1,000 V/cm + 5 mmol/L Met, the ROS levels exponentially increase to 9.6-fold with respect to the control, indicating synergy.

Tukey’s multiple comparison tests (Table 4) provide statistical insights into the differences in ROS levels among the different treatments. The control, 1,000 V/cm, 5 mmol/L Met, and 1,000 V/cm + 5 mmol/L Met are statistically different from each other (*P* value) and are represented by the letters “A”, “B”, “C”, and “D”, respectively.

**Table 4:** Summary of Tukey’s multiple comparisons test for ROS assay in MDA-MB-231 at 24 h

**Tukey’s multiple comparisons**	**Mean difference**	**95% CI of difference**	**Above HSD**	**Summary**	**Adjusted *P* value**
Control *vs.* 1,000 V/cm	–1,120	–1,488 to –752	Yes	****	< 0.0001
1,000 V/cm *vs.* 5 mmol/L Met	–3,120	–3,489 to –2,752	Yes	****	< 0.0001
5 mmol/L Met *vs.* 1,000 V/cm + 5 mmol/L	–3,611	–3,979 to –3,243	Yes	****	< 0.0001

^****^ *P* < 0.0001

### Wound healing assay

**Figure 9:**
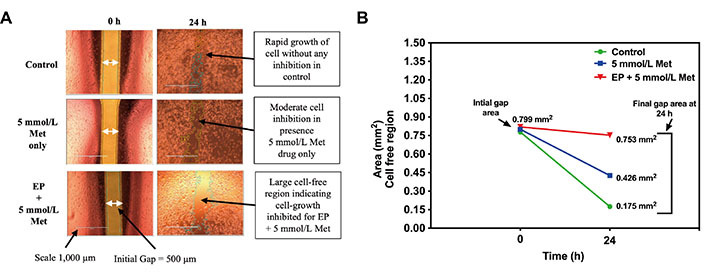
Wound healing assay in MDA-MB-231 cells at t = 0 h and t = 24 h for control, 5 mmol/L Met, 1,000 V/cm + 5mmol/L. (A) Microscopic image; (B) cell-free area (mm^2^) quantified using ImageJ

The microscopic images of wound healing assay inspection for control, 5 mmol/L Met, and 1,000 V/cm + 5 mmol/L Met at t = 0 h and t = 24 h are shown in Figure 9. The images at t = 0 h in Figure 9A were taken after the IbidiTM culture-insert 2-well was removed to see a cell-free gap of 500 µm in each sample. After incubation, the images at t = 24 h show that the control specimens proliferate without any inhibition, almost filling the gap. It shows the unrestricted migration of MDA-MB-231 cells. The 5 mmol/L Met treated sample inhibits cell migration and exhibits reduced migration compared to the control. Further, 1,000 V/cm + 5 mmol/L Met showed a more significant reduction in migration than the 5 mmol/L Met and control.

The cell-free area (in mm^2^) at t = 0 h and t = 24 h obtained from the ImageJ analysis is shown in Figure 9B. At t = 0 h, all the sample treatments (control, 5 mmol/L Met, 1,000 V/cm + 5 mmol/L Met) exhibited similar cell-free areas of 0.778 mm^2^, 0.800 mm^2^, and 0.820 mm^2^, respectively. However, at t = 24 h, a notable divergence in the control group displayed a reduced cell-free area of 0.175 mm^2^, indicating substantial migration and wound closure. In contrast, the 5 mmol/L Met group showed a moderately reduced cell-free area of 0.426 mm^2^, suggesting a potential impairment in cell migration and wound closure. The 1,000 V/cm + 5 mmol/L Met group exhibited the largest cell-free area of 0.753 mm^2^, signifying a pronounced inhibition of cell migration.

## Discussion

**Figure 10:**
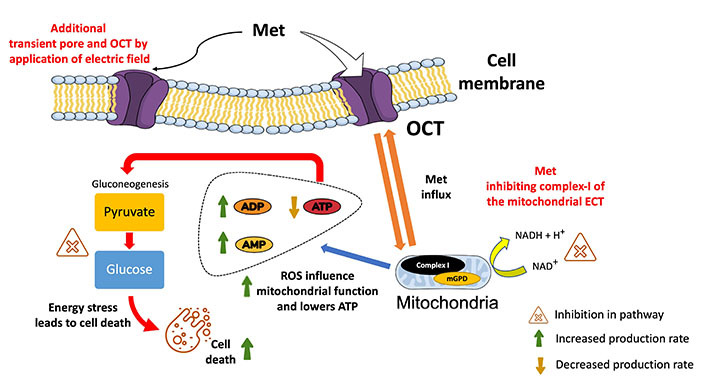
Illustration of the overview of the plausible mechanism of action for Met in the presence of electric pulses. It shows the effective entering of Met into the cell via the OCT and additional pores created by EP (at 1,000 V/cm). Met promotes the conversion of ATP to adenosine diphosphate (ADP) and AMP, thereby lowering mitochondrial energy production. Met’s impact on gluconeogenesis is highlighted by showing the inhibition of converting pyruvate to glucose. By impeding this process, Met contributes to decreased glucose levels and elevated ROS levels, creating an overall energy stress situation within the cell, ultimately leading to cell death. mGPD: mitochondrial glycerol-3-phosphate dehydrogenase; NAD^+^: depleted nicotinamide adenine dinucleotide

Met enters cells through a process called transporter-mediated uptake. The primary transporter responsible for Met uptake is the organic cation transporter (OCT), which is expressed on the cell membrane (Figure 10) [88]. Prior studies have shown that EP can increase the uptake of external molecules up to 1,000 times (using bleomycin) through transient permeabilization of the cell membrane [89, 90] and by increasing the expression of transporters like OCT [91, 92]. This suggests a greater drug uptake for the combined treatment. Also, it is a favorable outcome for repurposing Met in the presence of EP as an anticancer agent for TNBC patients.

The microscopic inspection of cells also corroborates the antiproliferative effects of combined treatment. Here, the cell confluency and deviation from the characteristic spindle-like shape were used to conclude the impact of different treatments on untreated MBA-MB-231 cells. For instance, cell confluency measures how much of the surface of a culture dish is covered by cells [93]. It is an indicator of cell growth and proliferation, and for 1,000 V/cm + 5 mmol/L Met, a more pronounced reduction in cell confluency indicates that cells are dying or not dividing as rapidly. Similarly, when MBA-MB-231 cells die, they lose their spindle-like shape, indicating cell death or cycle arrest [94]. As seen in the case of 1,000 V/cm + 5 mmol/L Met, the observed sizable circular dead cell debris, suggesting enhanced cytotoxic effects of Met in the presence of EP (1,000 V/cm + 5 mmol/L Met), inducing cell cycle arrest.

Further, wound healing assay used for chemotaxis assessment (the migration of cells under the influence of a drug) [95] also indicates the same reduction in cell proliferation. It measures cell migration by creating a wound in a monolayer of cells and measuring how quickly the wound closes. The cell migration is directly correlated to the metastatic potential of cancer cells [96]. A faster wound closure rate indicates that the cells are migrating and proliferating more rapidly. In contrast, inhibition by decreased wound closure into the scratched area indicates the treatment’s capability to hinder the invasive potential of TNBC cells [97]. The results indicated that the combined treatment by Met in the presence of electric pulses (1,000 V/cm + 5 mmol/L Met) significantly impairs cell migration and inhibits wound closure. It is a positive outcome, as reduced cell migration is directly associated with decreased metastatic potential in cancer cells.

The working mechanisms of Met in cancer are complicated, and exploring various techniques to comprehend the pathways is still an ongoing task [98]. In general, TNBC cells display altered glucose metabolism by overexpressed glucose transporters [99] and exhibit dysregulation in glucose uptake by relying heavily on glucose for energy production (glycolysis) [100, 101]. The glucose assay results showed a 5.6-fold reduction in glucose levels for combined treatment (1,000 V/cm + 5 mmol/L Met) compared to the control. It suggests modulation in glucose metabolic pathways leading to low adenosine triphosphate (ATP) energy formation and enhanced cell death [102].

Glucose metabolic pathways are vital for maintaining cellular energy homeostasis [103]. Among the key players in these pathways are GLUT2, which facilitates glucose transport into cells, and glucokinase (GCK), catalyzing the initial step of glycolysis [104]. Phosphofructokinase 1 (PFK1) is a central glycolysis regulator, while pyruvate kinase M2 (PKM2) catalyzes the final glycolysis step, leading to pyruvate generation [105]. Furthermore, oxidative phosphorylation proteins (OXPHOS) are responsible for generating ATP from nicotinamide adenine dinucleotide (NADH) and flavin adenine dinucleotide (FADH2) in the electron transport chain (ETC) [106]. Next, glucose-6-phosphate dehydrogenase (G6PD) initiates the pentose phosphate pathway, while glucose-6-phosphatase (G6PC) catalyzes the initial step of gluconeogenesis [107]. These pathways are crucial for producing energy (ATP) and, if hindered, lead to glucose lowering, creating energy stress and deficiency [108] as shown in Figure 10.

Subsequently, Met has been shown to disrupt glucose homeostasis in cancer cells [109] by inhibiting complex I of the mitochondrial ETC and impairing ATP synthesis. This leads to energy deprivation and compromising vital cellular functions [110]. Firstly, due to a higher influx of Met in the presence of electric pulses, as shown in Figure 10, inhibition of complex I of the mitochondrial ETC decreases ATP production (from NADH) by preventing the transfer of electrons to subsequent complexes [111]. This decreases the proton gradient across the inner mitochondrial membrane, which is required for ATP synthesis. Subsequently, the increase in AMP and ADP combined with lower levels of ATP inhibits the enzymes needed for gluconeogenesis (a process generating glucose from non-carbohydrate sources).

Secondly, it increased ROS production. When complex I is inhibited by Met, electrons leak from the ETC to oxygen, forming ROS [112]. ROS imbalance can damage cellular components, such as DNA, proteins, and lipids, due to oxidative stress-mediated cell death pathways [113]. ROS include molecules like H_2_O_2_, which are generated as natural byproducts of cellular metabolism and essential proper functioning of living cells [114]. However, the experiment shows an upregulation (9.56-fold with respect to control) in ROS levels with Met treatment in the presence of EP. Prior studies show that Met has the ability to boost cell ROS levels [115], creating an imbalance between the production of ROS and the cell’s ability to detoxify them or repair the resulting damage [116]. ROS influence’s mitochondrial function, as excessive levels can damage mitochondrial components and impair oxidative phosphorylation, leading to altered energy production and cellular metabolism [117, 118].

Oxidative stress-mediated cell death pathways involve a complex interplay of genes and proteins responsible for cellular component damage [119, 120]. The tumor protein 53 (p53) is a prominent regulator, functioning as a transcription factor that governs the expression of genes involved in cell cycle arrest, apoptosis, and DNA repair [121]. Additionally, pro-apoptotic proteins such as B-cell lymphoma 2 (Bcl-2)-associated X protein (Bax) and Bcl-2 homologous antagonist killer (Bak) operate as effectors in the intrinsic apoptotic pathway, forming pores in the mitochondrial membrane, thereby facilitating the release of cytochrome c and triggering apoptosis [122]. All this evidence suggests that excessive ROS production with Met treatment in the presence of an electric field inhibits MDA-MB-231 cell growth due to oxidative stress.

Overall, the results indicate the impact of the various interventions, including electroporation, Met, and its combination, on cell viability, glucose, ROS, and cell migration in the MDA-MB-231 cells. The synergy of EP and Met is indicated a viability as low as 42.60% at 24 h for MDA-MB-231 cells. They also show the enhanced efficacy of Met in the presence of EP, disrupting glucose and ROS levels and hindering TNBC cell proliferation and survival. Thus, these studies lay a strong foundation for developing novel therapeutic approaches to treat TNBC patients using Met and contribute to the growing knowledge in TNBC research.

The limitations of this research include the use of *in vitro* model, under controlled laboratory conditions. This only mimic partially the true *in vivo* tumor microenvironment. However, the preliminary results provide the potential for several avenues of future research, such as the possibility of clinical translation (preclinical animal models and clinical trials) and the study of long-term effects, including developing potential resistance mechanisms.

## Abbreviations

ADP: adenosine diphosphate
AMP: adenosine monophosphate
ANOVA: analysis of variance
ATP: adenosine triphosphate
BRCA1: breast cancer gene 1
CI: confidence interval
EP: electrical pulses
ETC: electron transport chain
HSD: honestly significant difference
Met: metformin
mTOR: mammalian target of rapamycin
ns: not significantly
OCT: organic cation transporter
RLU: relative luminescence units
ROS: reactive oxygen species
TNBC: triple-negative breast cancer

## Declarations

### Availability of data and materials

The raw data supporting the conclusions of this manuscript will be made available by the authors (Raji Sundararajan, raji@purdue.edu), without undue reservation, to any qualified researcher.

### Copyright

© The Author(s) 2024.
